# Highlights in soft tissue sarcomas and gastrointestinal stromal tumours (GIST) trials reported at ASCO 2017 Annual Meeting

**DOI:** 10.1186/s12916-017-0931-4

**Published:** 2017-08-22

**Authors:** Anna Maria Frezza, Silvia Stacchiotti, Alessandro Gronchi

**Affiliations:** 1 Department of Cancer Medicine, Fondazione IRCCS Istituto Nazionale dei Tumori, Milan, Italy; 2 Department of Surgery, Fondazione IRCCS Istituto Nazionale dei Tumori, Via Venezian 1, 20133 Milan, Italy

**Keywords:** Sarcoma, Gastrointestinal stromal tumours, Prognostic assessment, Preoperative treatment, Chemotherapy, Antiangiogenic drugs, Immunotherapy, Next generation sequencing

## Abstract

Herein, we summarise the results of the most relevant studies presented at the 2017 ASCO Annual Meeting in the field of soft tissue sarcomas (STSs) and gastrointestinal stromal tumours (GISTs). Innovations on the management of localised disease, highlights from the different experiences in the metastatic setting and large studies on rare histologies will be included. Special attention will be paid to results on immunotherapy, antiangiogenics use in histology with limited sensitivity to standard chemotherapy and new compounds. The preliminary results on the impact of the next generation sequencing in the everyday management of STS and GIST patients will be also discussed.

## Localised disease: prognostic stratification and treatment

The “Sarculator”, a nomogram designed to improve prognostic assessment in localised soft tissue sarcomas (STSs), was used for the further prognostic stratification, within a selected population, of “high-risk” STS patients treated with preoperative chemotherapy in a randomised clinical trial (RCT) [1]. The low, intermediate and high-risk groups identified with the Sarculator were characterised by the distinct cumulative incidence of distant metastases (0.26, 0.31 and 0.48, respectively) and overall survival (OS) rates (0.78, 0.63 and 0.42, respectively). Therefore, as highlighted at the ASCO 2017 Annual Meeting, the Sarculator should be considered to redefine high-risk STS and to stratify patient populations for future trials investigating perioperative chemotherapy. A full-dose anthracycline-based regimen is the current preoperative treatment of choice for STS. Trabectedin has a distinct activity in myxoid round cell liposarcoma (MRCL), which is also sensitive to radiation therapy (RT). A European phase I study confirmed the feasibility of a combined preoperative treatment with a short-course of trabectedin and concurrent low-dose RT in patients with locally advanced MRCL [2]. A trabectedin dose of 1.5 mg/m^2^ was the recommended phase II dose, with all patients completing RT. Interestingly, 75% of patients achieved a good pathological response (≤10% viable tumour), and 25% achieved a complete response.

PERSIST-5 [3], a phase II single-arm study, investigated the value of extending post-operative treatment for gastrointestinal stromal tumours (GISTs) with imatinib for up to 5 years in 91 patients with high risk of recurrence and confirmed imatinib effectiveness in preventing recurrence in patients with sensitive mutations; among seven relapsing patients, only one progressed (D842V mutation). Nevertheless, approximately 50% of patients discontinued treatment early, and most recurrences occurred after imatinib discontinuation.

## Advanced disease: chemotherapy, antiangiogenic drugs and newer compounds

Aldoxorubicin, a prodrug of doxorubicin, binds natural albumin on entry into the bloodstream. In hypoxic regions, such as the cancer environment, the linker between doxorubicin and albumin is hydrolysed, leading to the release of active doxorubicin. A phase III RCT evaluated the efficacy and safety of aldoxorubicin compared to investigators’ choice of treatment in 333 pretreated STS patients, 66% of whom had prior exposure to doxorubicin. The study showed a favourable trend in progression-free survival (PFS) and disease control rate for aldoxorubicin (4.11 vs. 2.96 months, *P* = 0.09; 34% vs. 25%, *P* = 0.06), which was statistically significant in the subgroup of liposarcoma/leiomyosarcoma (5.32 vs. 2.96 months, *P* = 0.007; 42% vs. 27%, *P* = 0.02) [4]; no difference was reported in OS. Aldoxorubicin was associated with minimal cardiotoxicity (up to 40 cycles) compared to doxorubicin and with a significantly lower incidence of alopecia. Encouraging data on the activity and safety of aldoxorubicin in combination with 14-day continuous infusion ifosfamide were also presented [5].

For STS subtypes known to be poorly responsive to conventional chemotherapy, antiangiogenic drugs are becoming a valuable treatment alternative. CASPS is a phase II, placebo-randomised study exploring the activity of cediranib in alveolar soft part sarcoma, an exceedingly rare STS subtype, indolent by nature and affecting young patients [6]. The results of this study, currently the largest on this subtype (48 patients), confirmed activity of the drug compared to placebo (risk ratio (RR) at 24 weeks: 21% vs. 0; *P* = 0.053), even in patients previously treated with other tyrosine kinase inhibitors. An improvement in PFS (10.8 vs. 3.7 months; *P* = 0.041) and OS at 12 months (96% vs. 64.3%) was reported. Similarly, the GEIS-32 single arm, phase II study explored the activity of pazopanib in patients with malignant/dedifferentiated solitary fibrous tumours and extraskeletal myxoid chondrosarcoma (EMC) [7]; the solitary fibrous tumour cohort (34 patients, 31 evaluable for response) showed a median PFS of 5.53 months, with size, mitoses and evidence of dedifferentiation being associated with a worst outcome. Choi criteria were shown to better capture treatment response compared to RECIST (RR: 52% vs. 3%) and, in multivariate analysis, were shown to be an independent predictor of OS. Preliminary results of the EMC cohort showed that, despite a modest activity reported for pazopanib (RR 5%), prolonged disease stabilisation occurred in a significant proportion of EMC patients, supporting further studies exploring pazopanib use in this disease [8]. In a phase II study with regorafenib including 30 pre-treated patients affected by Ewing sarcoma and related tumours [9], only three achieved a partial response, with a median PFS of 6 months. Interestingly, one of the responding patients carried the CIC-DUX4 translocation; thus, the Ewing variant without EWSR1-FLI1 fusion might benefit more.

Epithelioid sarcoma (ES) is poorly sensitive to chemotherapy. A large, international retrospective series including advanced ES patients treated with anthracycline- and gemcitabine-based regimens was presented [10], with a drug activity (RR: 25% and 23%, respectively) similar to that reported in other adult STS. In this series, the largest available, the value of pazopanib seemed limited. ES is marked by the loss of INI1, detected in more than 90% of cases. Tazemetostat, an oral selective EZH2 inhibitor, has shown activity in INI1-deleted solid tumours, including ES [11]. In a phase II study including 31 ES patients [11], four showed partial response and two had prolonged stable disease, with a favourable drug tolerability. Following these results, enrolment has been expanded to 60 patients, with accrual recently completed.

A major breakthrough in the field of advanced GIST was achieved by a phase I study assessing the safety and activity of BLU-285 in refractory or primary PDGFRα D842V-mutated GIST [12]. BLU-285 is a selective oral inhibitor that targets KIT Exon 17 and PDGFRα D842 activation loop mutants, reported to be well tolerated and associated with a RR of 18% in refractory KIT patients. In patients harbouring the D842V mutation, for whom no active systemic treatment is available, the RR was 41%, with responses across all dose levels.

## Immunotherapy: PD1/PDL1 inhibitors and anti-NY-ESO-1 regimens

The activity of different anti-PD1/PDL1 antibodies has been explored in STS and GIST. A non-comparative phase II study randomised 85 refractory STS patients to receive nivolumab or nivolumab + ipilimumab [13]. Minimal activity was observed with nivolumab alone (RR 5%), whereas the combination with ipilimumab showed favourable safety and promising antitumor activity (RR 16%), with responses recorded in multiple subtypes. Similarly, the phase II study SARC028 explored the activity of pembrolizumab in 86 STS and bone sarcoma [14]. A RR of 40 and 20% was reported for undifferentiated pleomorphic sarcoma and dedifferentiated liposarcoma, respectively; expansion cohorts in those subtypes are planned. PD-L1 expression in pre-treated tissue was uncommon (4%), but correlated with T-cell infiltration and response in undifferentiated pleomorphic sarcoma. The combination of pembrolizumab and oral cyclophosphamide has been tested in STS and GIST through a phase II study from the French Sarcoma Group [15]; however, from over 50 evaluable patients, only one response was reported.

Interesting results have also been reported with anti-NY-ESO-1 T-cells [16]. NY-ESO-1 antigen is expressed in approximately 20% of adult STS of all types, with a higher incidence in specific subtypes [17]. NY-ESO-1 positivity was found to correlate with favourable PFS on standard treatment. Twenty-five STS patients received CMB305 (an active immunotherapy regimen which generates and expands anti-NY-ESO-1 T-cells) within a phase I study testing the safety, immunogenicity and efficacy of the drug in NY-ESO-1-positive solid cancers, with the results proving a favourable tolerability profile [18]. Among STS, 64% of patients developed NY-ESO-1-specific T-cells and 72% developed anti-NY-ESO-1 antibodies. The 3-month PFS was 74 and 75% for synovial sarcoma and MRCL, with a 1-year OS rate of 86 and 100%, respectively. A RCT of CMB305 in combination with atezolizumab in synovial sarcoma and MRCL patients is ongoing [18].

STS results from the studies presented, including responsive histologies, are summarised in Table 1.

**Table 1:** Immunotherapy in soft tissue sarcoma – results from the 2017 ASCO Annual Meeting

Study	Regimen	Patients evaluable for response	ORR^a^	Complete response (histology)	Partial response (histology)	Stable disease	Progressive disease	Median duration of response (weeks)
Burgess et al. [14]	P, 200 mg i.v., Q3 weeks	STS: 40	STS: 18%	1 (UPS)	6 (3 UPS, 2 DDLPS, 1 SS)	15	18	33
		BS: 40	BS: 5%	0	2 (1 OS, 1 CS)	9	29	43
Toulmonde et al. [15]	P, 200 mg i.v., Q3 weeks; oral cyclophosphamide 50 mg alternative weeks	50	2%	0	1 (NA)	NA	NA	NA
D’Angelo et al. [13]	N, 3 mg/kg, Q2 weeks	N: 38	N: 5%	0	3 (ASPS, LMS, sarcoma NOS)	15	20	NA
	N, 3 mg/kg, Q3 weeks for 4 cycles, then Q2 weeks; I: 1 mg/kg Q3 weeks for 4 cycles	N + I: 38	N + I: 16%	2 (MFS, uterine LMS)	4 (3 UPS, LMS, angiosarcoma)	19	10	NA
Somaiah et al. [18]	CMB305 regimen (LV305 i.d. injections alternating with G305 i.m. injections for 3 months, then bimonthly G305 injections up to 1 year)	25	0	0	0	16	9	NA

*ASPS* alveolar soft part sarcoma, *BS* bone sarcoma, *CS* chondrosarcoma, *I* ipilimumab, *DDLPS* dedifferentiated liposarcoma, *LMS* leiomyosarcoma, *MFS* myxofibrosarcoma, *N* nivolumab, *NA* not available, *NOS* not otherwise specified, *ORR* overall response rate, *OS* osteosarcoma, *P* pembrolizumab, *SS* synovial sarcoma, *STS* soft tissue sarcoma, *UPS* undifferentiated pleomorphic sarcoma

^a^RECIST 1.1

## Next generation sequencing (NGS) in the management of sarcoma and GIST

**Fig. 1:**
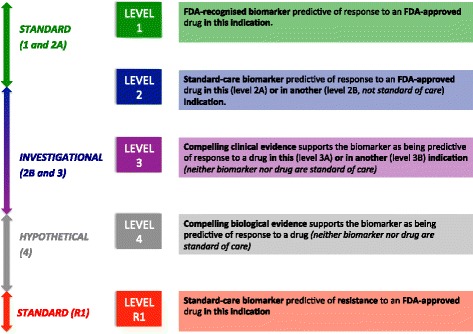
OncoKb Level of Evidence system [22] (adapted version). Each mutational event is assigned with a level of clinical actionability (ranging from standard-of-care to investigational or hypothetical treatments)

The value of NGS in the diagnosis and identification of new targets for STS and bone sarcoma is yet to be elucidated; however, results from the two largest retrospective studies were reported at the 2017 ASCO Meeting. In the series from Gounder et al. [19], 5635 sarcoma patients underwent genomic profiling worldwide. A modest impact of NGS was observed in aiding diagnosis (only 8% of initial pathological diagnoses changed). Conversely, ‘actionable’ mutations were identified in 57% of tested cases. According to the OncoKB system (Fig. 1), 8% were level 1 evidence, 10% level 2 and 39% level 3. Similarly, in the French retrospective experience of 587 STS patients [20], 93% had at least one targetable mutation, copy number alteration and/or fusion gene. The significant proportion of actionable alterations detected in these two series could lead to the implementation of NGS for clinical decision-making in patients with advanced STS. A phase III study testing the superiority of NGS-guided treatments compared to a conventional strategy in STS patients refractory to anthracycline (MULTISARC study) is being discussed. NGS routine testing has also been reported as informative in the management of GIST. Kelly et al. [21] reported on 117 prospectively tested patients; NGS was found to influence clinical decision-making in 79% of patients and actionable mutations were identified in 81%; thus, the development of a GIST-specific panel might be of interest.
